# Use of E-Beam for Shelf-Life Extension and Sanitizing of Marinated Pork Loin

**DOI:** 10.1155/2012/962846

**Published:** 2012-11-21

**Authors:** I. García-Márquez, J. A. Ordóñez, M. I. Cambero, M. C. Cabeza

**Affiliations:** Departamento de Nutrición, Bromatología y Tecnología de los Alimentos, Facultad de Veterinaria, Universidad Complutense de Madrid, 28040 Madrid, Spain

## Abstract

The effectiveness of E-beam radiation to extend the shelf-life of marinated pork loin slices stored at 4 and 8°C (temperature abuse) has been studied. The shelf-life was extended from 7 to 16 and >20 days after the application of 1 and 2 kGy, respectively. In the event of a temperature abuse occuring during the product distribution (e.g., increase to 8°C), the shelf-life would be extended from 5 to 10 and 16 days, respectively, when applying the doses mentioned previously. From a public health point of view, the irradiation of marinated pork loin may be marketable for a longer period of time of up to two weeks, and guarantees a practically *Salmonella* and *Listeria*-free product. Minor changes are produced by the E-beam treatment in the main sensory and rheological characteristics. The odor was the most affected feature, but the off-odors diminished with increased storage. In any case, testers judged the samples to be adequate for marketing.

## 1. Introduction

In order to diversify the meat products consumed by the population, the meat industry has implemented marinating technology to help satisfy the psychological need of consumers to broaden their choice of foods and to maintain its market share. Several studies have been carried out to optimize this technology in different kind of meats, namely, beef, pork, and poultry [1–5] although, according to the authors of former papers, marinated pork is, perhaps, the most common. The marinating is based on the water-binding capacity of several compounds, such as sodium chloride, sodium lactate, calcium lactate, lactic acid, and calcium chloride. The salt plays several functions and provides some functional properties to the meat. As a bacteriostatic agent, the salt increases the shelf-life of meat and meat products by reducing the growth rate of spoilage bacteria [6, 7]. Similarly, the salt activates the protein to increase hydration and its water holding capacity, thus increasing the binding properties of proteins, which is an important effect since the meat proteins can swell to twice their original size [7]. Additionally, salt improves the texture [8, 9] and palatability [1] by increasing its juiciness [10], and it also improves the tenderness and overall acceptability [11, 12]. The addition of sodium lactate was shown to improve color and to help to stabilize this feature during storage [13–15].

Fresh meat presents a highly variable microbiota [16, 17] but regardless of the initial number of organisms, the most common spoilage of fresh meat in air-stored conditions is dominated by Gram-negative, psychrotrophic, aerobic rod-shaped bacteria. Although a wide range of genera are present on meat, only *Pseudomonas*, *Acinetobacter*, and *Psychrobacter* (formerly some *Acinetobacter* and *Moraxella*) species are normally considered to be important [18]. Of these, *Pseudomonas* species are of greatest concern [19, 20]. *Pseudomonas* spp. typically account for >50% of the total microbiota and sometimes even up to 90% [18]. The spoilage is manifested by the release of off-odors when the number of bacteria reaches the level of about 5 × 10^7^ CFU/cm^2^ and the appearance of slime when the bacterial load reaches the value 10^8^ CFU/cm^2^ [21, 22]. 

Marinated meat products have some of the benefits discussed above, but they may have an added problem and limited food safety, since there may be pathogenic cross-contamination during processing and storage, as well as from raw materials such as fresh meat, sauce, and fresh vegetables [23, 24].

Marinated pork is mainly distributed by the industry as whole pieces but they are usually displayed as slices packed in family-sized portions exposed on the refrigerated shelves of supermarkets so that consumer can freely choose the product, which is then cooked at home. The slices preparation involves manipulation during which the product may be potentially contaminated with pathogen bacteria from the environment, handlers, equipment, and so forth. An additional problem is the temperature abuse (e.g., increase to 7–10°C), which could occur during distribution. Among the pathogens that may be encountered, those of the greatest concern are* Listeria monocytogenes* and *Salmonella* spp. They can be considered ubiquitous. In fact, they are frequently detected in a variety of foods [16]. However, *L. monocytogenes* is the most dangerous of the two organisms since it is a facultative anaerobic, which grows even under strict refrigeration [25, 26], while *Salmonella* spp. are not able to multiply at refrigerated temperatures but they can at >7°C [26, 27]. Another highly dangerous pathogen is *Escherichia coli* O157:H7 since it is responsible for a severe disease process [26, 28]. However, it is less frequent and unable to grow under refrigerated conditions but, like *Salmonella* spp., it can multiply when there is an uncontrolled increase in temperature. Thus, the risk of the food-borne pathogens growth may be higher when there is a temperature abuse by retailers and consumers [29].

The shelf-life of fresh pork loin is very short. It does not last any longer than 5–7 days under refrigeration [30]. It may be considered that the marinated pork also has a short shelf-life since this is an uncooked product. Considering to the current trade situation of perishable foods over long distances, it is obvious that any action applied to increase the shelf-life of this product would be very useful from a commercial point of view. The present work proposes the treatment with accelerated electrons (E-beam) to extend its shelf-life. This treatment is also useful to substantially reduce the potential pathogens that may be present since that E-beam is an effective procedure to kill vegetative pathogen bacteria [31–36].

## 2. Materials and Methods

### 2.1. Organisms

*Salmonella* spp. resistance against E-beam radiation is similar to that of *L. monocytogenes* [37, 38], and it has been repeatedly demonstrated that *E. coli* O157:H7 is more radiosensitive than *L. monocytogenes* [31, 33, 37]. Therefore, the results obtained for the *L. monocytogenes* may also be extended to two other species with a high confidence range (95%) due to the psychrotrophic character of *L. monocytogenes. *This feature must be kept in mind to optimize the treatment and research objectives. Likewise, in previous works, we have observed [34] that *L. innocua* NCTC 11288 is more radioresistant than five strains of *L. monocytogenes*. Accordingly, *L. innocua*, as a surrogate of *L. monocytogenes*, was used as the target organism for experiments.

*L. innocua* NCTC 11288 was supplied by the Spanish Type Culture Collection (CECT, Valencia University, C. Dr. Moliner s/n. Burjassot, 46100. Valencia. Spain). Fresh cultures were prepared by removing a piece of frozen culture and inoculating it into trypticase soy broth, then incubating at 32°C for 24 h. The culture was subsequently centrifuged at 4°C and the pellet suspended in a sterile test tube with 10 mL sterile saline, which yielded a bacterial load that was close to 10^8^ cells/mL. The handling, subculture, and inoculum preparation of *L. innocua* and sample contamination have been previously described for other meat product slices [34, 35].

### 2.2. Sample Preparation and Irradiation Treatment

Marinated pork loins were prepared in a local industry under supervision of one of the authors. Whole loins were placed in a rotating drum, in which they were massaged for 15 min in a brine consisting of (w/v) salt (1.6%), nitrates and nitrites (0.025% of KNO_3_/NaNO_2_ (2/1) (w/w)), sodium ascorbate (0.080%), and spices (1.4% of a mixture of white pepper/paprika (2/12) (w/w)). Then, they remain submerged in the brine for 2 days at 2–4°C. Slices (4–6 mm thickness) were cut from freshly marinated pieces (3-4 Kg in weight) using an electric machine, whose rotating blade and contact surfaces were previously deeply cleaned with hot water (around 90°C) and ethanol 70°. Following this, 2–4 slices were packaged in low permeability plastic (60 *μ*m copolymer of poliamide/polietilene) bags (diffusion coefficient of 35 cm^3^/24 h m^2^ bar to oxygen and 150 cm^3^/24 h m^2^ bar to carbon dioxide) and heat-sealed without air exclusion. The gases ratio for filling bags was air/meat 4/1 (volume bag/sample weight). Samples were transported to the irradiation plant in refrigerated boxes. They were treated in an industrial electron beam radiation source, which operates at 10 MeV located in Tarancón, Cuenca, Spain, 60 km from the laboratory. The radiation doses employed were between 0.2 and 3 kGy, namely, 0.2, 0.5, 1, 1.5, 2, 2.5, and 3 kGy and the dose absorbed by samples was verified considering the absorbance of cellulose triacetate dosimeters [39] simultaneously irradiated. 

To determine the inactivation of *L. innocua*, slices were contaminated by immersion for 10 sec in the cell suspension (higher than 10^8^ cells/mL formerly described [34], which yield an initial load of approximately 10^8^ CFU/cm^2^. A large number of cells (>10^7^ cells/cm^2^) were used to determine the death kinetic parameters accurately. The contaminated (one slice per trial for microbial analysis) and uncontaminated (four-five slices for physicochemical or sensorial analysis) samples were packaged as mentioned above. Experiments were performed at room temperature (18–20°C) by triplicate. The temperature increase during treatment was less than 2°C. Following the irradiation treatment, samples were transported (less than 1 h) in insulated boxes to the laboratory and they were stored in termostated chambers at 4 and 8°C, the latter was used as an example of temperature abuse during product distribution.

### 2.3. Microbial Analysis

To count the survivors, an aliquot (about 1 g) were homogenized with 10 mL of a sterile saline solution in a Stomacher bag. Total viable counts (TVC) were determined by the pour-plate method using Plate Count Agar (PCA; Difco, Detroit, MI, USA) containing 2% (w/v) NaCl [40, 41] as the culture medium. Plates were incubated for 48 h at 32°C. Lactic acid bacteria (LAB) were enumerated in double layer MRS agar (Conda-Pronadisa, Madrid, Spain) at pH 5.5 [42, 43]. The incubation was carried out at 32°C for 48 h. *Enteriobacteriaceae* were counted in violet red bile glucose agar (Oxoid, Basingstoke, Hampshire, UK) after an incubation of 24 h at 37°C.* Pseudomonas* spp. counts were determined after incubation at 25°C for 48 h in pseudomonas agar base, supplemented with cetrimide, fucidin, and cephalosporin (Oxoid), *Brochothrix thermosphacta* was enumerated in STAA (Oxoid) at 25°C for 48 h. The selective Palcam medium (Oxoid) for *Listeria* spp. was elected to assay the survival counts of this organism and to avoid the growth of endogenous microbiota. Colonies were enumerated with a Digital S Colony counter (J.P. Selecta, Barcelona, Spain). The growth curves were constructed according to the Baranyi model [44].

In shelf-life experiments, irradiated and nonirradiated loin slices were removed from the trays, in which several groups of organisms were periodically determined. From a microbiological point of view, the end of shelf-life was established when the total viable counts exceeded the value of 5 × 10^7^ CFU/cm^2^. Analyses were performed just after E-beam treatment (0 days) and at various times during storage until the end of the shelf-life.

### 2.4. Dry Matter, pH, and Water Activity (*a*_*w*_)

The dry matter was analyzed by the oven air-drying method (AOAC, 1995). The marinated loin pH was determined in a homogenate of the sample with distilled water (1 : 10) (w/v), using a Crison Digit-501 pH meter (Crison Instruments LTD, Barcelona, Spain). The *a*_*w*_ was measured using a Decagon CX1 hygrometer (Decagon Devices Inc., Pullman, WA, USA) at 25°C.

### 2.5. Water-Holding Capacity (WHC)

The WHC was measured by using the Carver Press Method [45]. The meat sample (0.3 g) was placed on a piece of filter paper (Whatman no. 1, 125 mm), then set between two plexiglas plates, and subjected to a mechanical force of 345 kPa for 5 min. The WHC values were calculated as the percentage of water retained based on water content in the product before pressing. Four replicates of each sample were determined.

### 2.6. Texture Measurements

The texture analysis were performed according to previous works [46, 47]. Texture tests were performed at about 22°C just after opening the bags. The TPA and tensile test were performed with a TA.XT2i SMS Stable Micro Systems Texture Analyser (Stable Microsystems Ltd., Surrey, England) using a cylindrical probe P/25 for TPA or a tensile grip (A/TGT) for the tensile test. The TPA was assayed in cylinders (1.5 cm high by 2 cm wide), and the tensile test was carried out on prismatic pieces (6 cm long, 2 cm wide, and 0.4 cm thick) of marinated loin samples. The resulting textural parameters were calculated as previously described by Herrero et al. [47]. 

### 2.7. Color Measurements

The measurements were performed using a tristimulus colorimeter (Minolta Chroma Meter CR300, Minolta Corporation, NJ). The values of the lightness (L*), redness (a*), and yellowness (b*) parameters were periodically (0, 5, and 10 days of storage at 4°C) measured 5–8 times on the surface of the E-beam treated and control (nontreated) slices at three different analyse times (0, 4, and 24 h to air exposure after opening the packaged bags). After the first color measurement, samples were kept at 6 ± 2°C and about 64 ± 2% relative humidity, without protection (similar conditions to a refrigerated display or a domestic refrigerator). Color parameters were determined in non-treated and treated samples at 0, 5, and 10 days of storage at 4°C.

### 2.8. Sensory Analysis

The sensory analyses involved a panel of twenty tasters (ten females and ten males) selected from the members of the Departamento de Nutrición, Bromatología y Tecnología de los Alimentos. Slices treated at 0, 1, and 2 kGy were used for sensory analysis. The following tests were performed: a triangular analysis, a rank order test, and a descriptive trial. The tests were carried out in individual booths built according to the International Standards Organization DP 66.58 [48] criteria. The analyses were carried out as described previously [49]. For the flavor analysis, pork loin steaks with a thickness of 0.5 cm were cooked during 2 min on each side using a grill-pan previously heated to 150°C. The temperature inside the steaks reached approximately 70°C, as measured by a portable digital thermometer (Testo model 735; Testo, S.A., Barcelona, Spain). This treatment was considered sufficient to obtain a good final degree of doneness. The appearance and odor were evaluated in raw and cooked samples. Only samples stored at 4°C were used for sensory analysis. The range order test was performed until the end of the shelf-life of untreated samples. Triangular and descriptive tests were performed until the end of the shelf-life of the untreated and treated samples.

### 2.9. Statistical Analysis

Survival curves were obtained by plotting the logarithm of the number of survivors against the dose assayed. Decimal reduction dose (*D*-values) were calculated from the linear regression equation of survival curves. Regression equations, coefficients of determination (*R* ^2^) and the error bars were calculated by Excel (Microsoft, Redmond, WA, USA). In the case of the data obtained with the physicochemical analysis, the differences among means were established by ANOVA and Duncan's multiple comparison procedure. These statistical analyses were performed using a Statgraphic Plus version 5.0 program. 

## 3. Results and Discussion

### 3.1. Physicochemical Characteristics

The fresh pork loin used in this research had average moisture content values of 74.16 ± 1.56%, an ash concentration of 1.88% ± 0.19%, dry matter content of 27.3 ± 1.6%, *a*_*w*_ = 0.992 ± 0.005, and pH = 5.71 ± 0.037. The effect of E-beam treatment (1 and 2 kGy) on the former parameters was negligible, except for WHC (Table 1). These results, excluding the latter feature, are in total agreement with previous data obtained for E-beam treated fresh pork loin [30]. The effect of radiation on the pH has been of concern of other authors. There were not observed effects in pork loin [50, 51] nor in ground beef patties [52] even when ascorbic acid was added to samples and they were treated with a dose as high as 10 kGy [52].

The effect of E-beam irradiation (1 and 2 kGy) on the WHC of the marinated product is recorded in Table 1. The slices showed a significant increase of the WHC (*P* < 0.05) just after the E-beam treatment. This result is completely opposite to what occurred in fresh loin, in which a decrease of the WHC was found [30]. Other authors [51, 53] have also reported a decrease in WHC, an increase in soluble protein, and exudates loss in irradiated pork *longissimus dorsi* muscle. These effects may be associated with changes produced by the irradiation in the muscle tissue structure, for example, an increase in the shrinkage of the myofibrils, as observed by Yoon [54] in chicken breast irradiated at 2.9 kGy. The WHC increase observed in marinated pork may be due to the absorption of exudates by the marinating compounds. This fact could be considered a positive effect since no fluids, or a lower quantity than in fresh pork, would be accumulated in the packaged bags.

### 3.2. Shelf-Life Aspects

**Figure 1:**
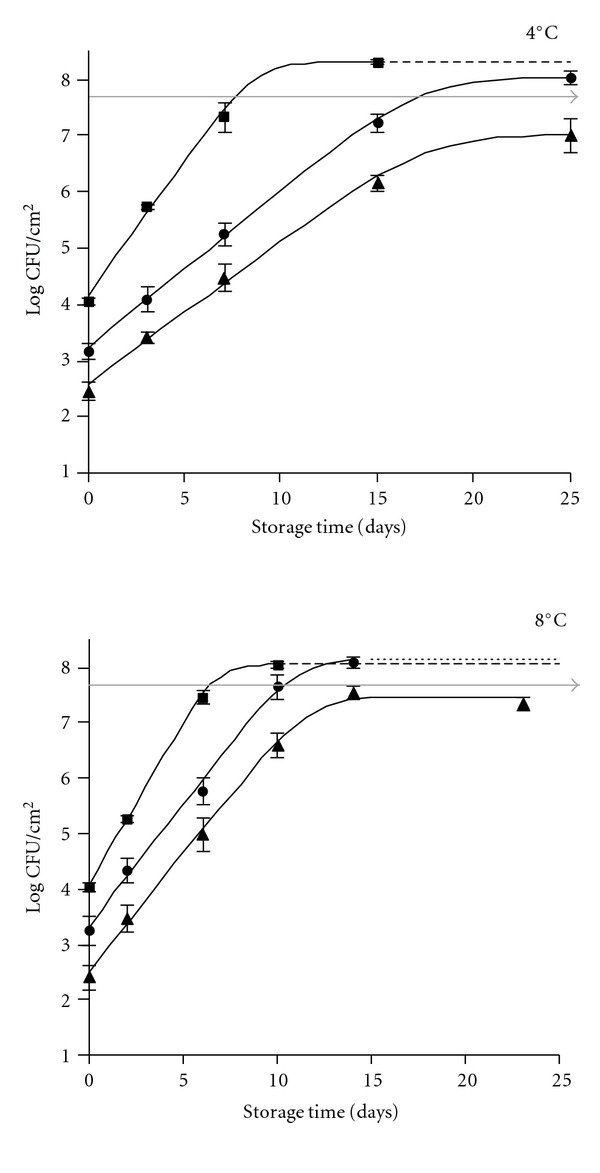
Changes in the total viable counts of marinated pork loin slices subjected to E-beam treatment and stored at 4°C and 8°C. Control (■), treated at 1 (●), and 2 kGy (▲).

Spoilage is a major concern in the food industry. In meat, the spoilage becomes apparent by the release of off-odors although there may be other phenomena such as color changes that also limit the shelf-life. To assay the effect of the E-beam application on the shelf-life of marinated pork loin, the changes in the total viable organisms in slices stored at 4 and 8°C (temperature abuse) for 25 days were studied (Figure 1). At 4°C, the initial total microbial count (TVC at day 0) of nonirradiated slices (control samples) was 4.0 log CFU/cm^2^. The treatment at 1 and 2 kGy caused a reduction of the bacterial number of about 1.6 and 2.2 log units, respectively. These data allow to approximately determine a *D*-value of about 0.90 kGy. This value is much higher than the one previously estimated in whole fresh loin [30]. The initial microbiota of raw intact meat is very complex. Usually, counts range from 10^3^–10^5^ CFU/cm^2^ [55, 56] but only 10% of the microbial population is able to continue growth upon refrigeration [57]. Both Gram-positive (*Micrococcus* spp., *Staphylococcus* spp., *Bacillus* spp., lactic acid bacteria, and the coryneform group) and Gram-negative (*Pseudomonas*, spp., *Shewanella* spp., *Psychrobacter* spp., and *Acinetobacter* spp.) bacteria have been detected [17]. Given this variety of bacteria, it is not easy to assert those microbial groups that are most affected by the radiation. The overall estimated *D*-value (0.90 kGy) was similar to the one reported by other authors for some vegetative bacteria, including lactic acid bacteria [58, 59] and the ubiquitous enterococci [60]. It is also close to that of the pathogens *S. aureus* [36, 61], *Salmonella* spp. [35, 61], and *L. monocytogenes* [34, 62] but higher than other pathogens, such as *Y. enterocolitica* and many gram-negative bacteria, in which *D*-values of 0.2–0.8 have been commonly reported [63–65]. According to the former values, it seems that the *D*-values of 0.90 kGy correspond to the radioresistance of Gram-positive bacteria. The Gram-negative bacteria were probably promptly reduced at very low levels and their survivors were unable to compete with the Gram-positive survivors. Therefore, the *D*-value only reflects the E-beam resistance of the Gram-positive bacteria. 

The initial total TVC determined in the fresh product (4.0 log units CFU/cm^2^) was in the range of the contamination reported by several authors [17, 55, 56]. The “lag phase” for TVC of nontreated samples was not observed at 4 nor at 8°C. Therefore, the natural microbiota began to multiply in the exponential growth phase, reaching the spoilage level (7.5 log units) after 7 and 5 days, respectively. The changes in the microbiota of the control samples during storage was in total accordance with Ayres' 1960 report [21], in which meat spoilage under refrigeration was exhaustively explained. A generation time (*g*-value) of 15 h was estimated at 4°C. Practically, the same pattern was observed in nontreated samples stored at 8°C, but, as expected, the *g*-value underwent a substantial decrease (*g*-value = 12.5 h) and, therefore, the shelf-life was shortened; it was estimated to be about 5 days. The shelf-life values were somewhat higher than those described by other authors [30, 66] in fresh pork. Therefore, the marinating compounds seem to have had an inhibitory effect on the indigenous microbiota. As the pH and *a*_*w*_ averages were in the level of those of fresh meat, some marinating compounds, namely, the species, may be responsible for the growth inhibition of the microbiota, which are endowed of antimicrobial effects [67], the Gram-negative bacteria being more sensitive than Gram-positive and LAB being the most resistant among the latter [68].

No characterization of the dominant microbiota was made but, according to the manifestation of spoilage (off-odor, putrid, cabbage), they most certainly were the aerobic spoilage organisms, that is, the Gram-negative bacteria, as has been described many times [17, 21, 69, 70]. The low permeability of the bags used in the experiments does not allow the rapid interchange of gases but even though oxygen is partially depleted by the microbial and residual tissue metabolism [71] the concentration of this gas in the bag is enough to allow the growth of aerobic bacteria at a growth rate similar to that observed in a nonrestricted atmosphere of air [70]. In fact, several studies have claimed that *Pseudomonas* spp. can grow in atmospheres of 1-2% of oxygen, even in presence of carbon dioxide [72].

The decrease caused in the initial bacterial load by the E-beam treatment resulted in a deceleration of its growth, which, in turn, led to a noticeable shelf-life extension (approximately a duplication) at 4°C since the value of 7.5 logs units was reached after 16 days with an estimated *g-*value of about 26 h. As expected, the same effects were observed when samples were stored at 8°C but a lower shelf-life extension was estimated (10 days). A similar model has been previously observed in fresh pork loin [30], and the shelf-life extension was attributed to both the lethal effect of E-beam and the deceleration of the growth rate of the surviving spoilage organisms. When doses of 2 kGy were applied, the shelf-life extension at 4°C was longer but at the end of the experiment (25 day) the level of log 7.5 CFU/cm^2^ was not reached. The former data allow to conclude that the application of a low dose of radiation is a useful procedure to attain an important significant shelf-life extension (16–25 days) even when a dose as low as 1 kGy is applied. These results may be of great importance from a commercial perspective since the marinated loin slices (and probably other anatomical regions of the carcass) may be displayed on the shelves of refrigerated cabinets for longer periods of times.

A lower degree of pork protection is achieved if a temperature abuse occurs since the surviving organisms will grow more rapidly and the time period during which the meat presents adequate conditions for consumption will be shorter. From the curves in Figure 1(b), *g*-values of about 18 h may be estimated regardless of whether the treatment was 1 or 2 kGy. The shelf-life at 8°C of E-beam treated marinated pork slices at 1 or 2 kGy was no longer than 10 or 15 days, respectively. The differences in the shelf-life were simply because the treatment of 2 kGy caused a greater reduction of the bacterial population and, therefore, the initial TVC was lower. Results indicate that E-beam treatment is also useful to extend the shelf-life for a significant period of time even at 8°C.  Table 2 shows a summary of shelf-life results. The marinated process leads to an increase in the shelf-life. However, the temperature abuse could originate public health problems because the higher temperature may promote the growth of pathogen organisms, if present, such as *Salmonella* spp. and *S. aureus,* since they are able to grow at 8°C but unable to grow at 4°C [26]. 

**Figure 2:**
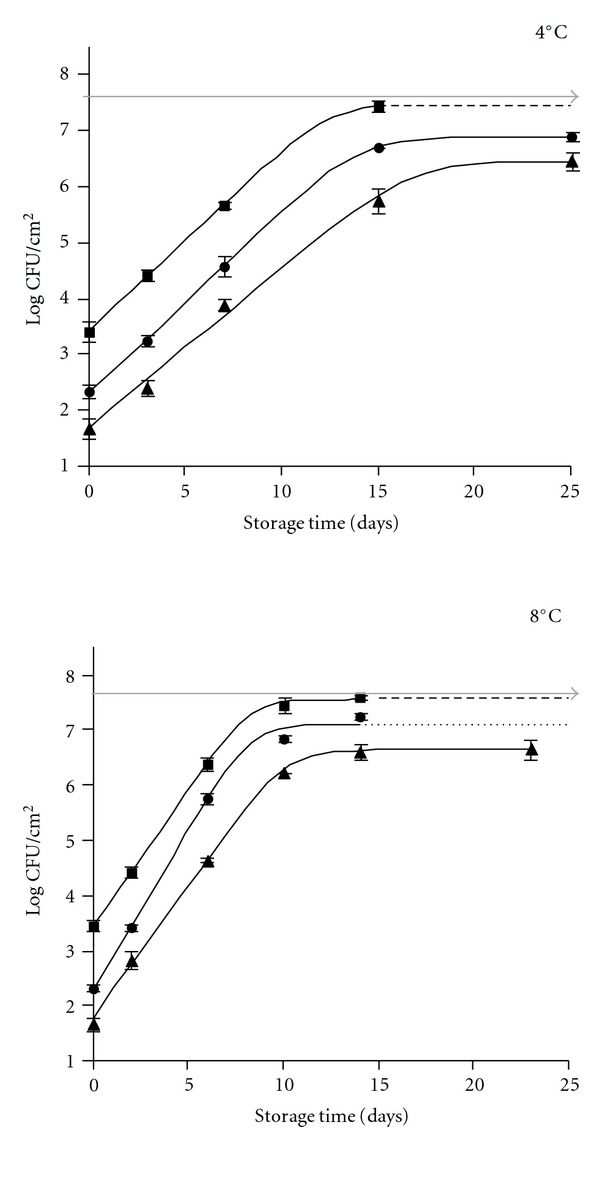
Changes in the lactic acid bacteria counts of marinated pork loin slices subjected to E-beam treatment and stored at 4°C and 8°C. Control (■), treated at 1 (●), and 2 kGy (▲).

The changes in the LAB counts during storage are shown in Figure 2. As expected, the E-beam caused a reduction in the initial number of LAB and a *D*-value of about 0.85 kGy was determined. It was higher than that obtained for TVC counts, which may be a consequence only LAB are involved in the counts since the medium used for counting (MRS agar) is selective for these organisms. Actually, the LAB are, among the nonsporeforming bacteria, the organisms with a high resistance to the ionizing radiation [58, 59]. These results are close to those reported by other authors for LAB in meat, since a treatment of 2.5 kGy produced only a 3.4 log reductions while more than five reductions were observed for other bacteria such pseudomonads, Enterobacteriaceae or *B. thermosphacta* [58]. The changes in the LAB during storage are noticeable because it is not frequent to observe these bacteria as a dominant group in the aerobically stored meat and, on the other hand, at both temperatures and at any treatment doses (0, 1, or 2 kGy) the behavior pattern was the same with the only difference being in the *g*-value. It was about 23 h at 4°C and 15 h at 8°C, which is considered logical since the greater the temperature the higher the growth rate. The LAB are the dominant organisms in marinated vacuum-packed pork at the expiration date [73], but it is difficult to explain the behavior of the LAB in the control slices under aerobic conditions. This behavior may be attributed to the carbon dioxide atmosphere enrichment as a consequence of the low permeability of the bag plastic. The microaerophile condition of the LAB is well known. Certainly, they have to compete with aerobic Gram-negative with a lower *g*-value (e.g., at 4°C, 15 h for TVC versus 23 h for LAB), which is reflected, for example, on day 15 at 4°C where the count of LAB was a log unit lower (10%) than that of TVC. In E-beam treated samples the circumstance described above coupled to the original very low level of Gram-negative bacteria since their numbers were severely reduced by the ionizing treatment and, therefore, the LAB (more radioresistant) have less organisms with which to compete. 

In control samples, the counts in the selective pseudomonas medium were very low in the first days of storage but by the 5th day log 5.0 CFU/cm^2^ colonies were counted and by the 7th day the level reach the value of log 7.5 CFU/cm^2^ (data not shown). The latter value is in total agreement with TVC data, which suggests that pseudomonads were the dominant organisms at the end of the shelf-life, as has been reported by other authors [17, 69, 70]. In E-beam treated (1 and 2 kGy) samples, it was not possible to monitor the changes in the pseudomonad population during storage. The counts in the selective medium for these organisms were not consistent, which has also been observed previously in fresh loin [30]. This was attributed to the selective substances (cetrimide, fucidin, and cephalosporin) added to the pseudomonas agar base to inhibit the growth of other organisms present in the samples. The E-beam may sensitize to pseudomonads, and then they were also inhibited by the supplement substances. In the case of 4°C, the temperature may act as an additional dysgenesic agent. 

*B. thermosphacta* and cold-tolerant Enterobacteriaceae bacteria also occur in aerobically-stored meat but because of their slower growth rate, they are poor competitors of the pseudomonads [18, 74]. In the present study, *B. thermosphacta* and Enterobacteriaceae were only occasionally found in control samples and at the end of the shelf-life, the former was seldom detected and the latter presented levels lower than log 4 CFU/cm^2^ at both 4 and 8°C. In irradiated samples, they were not detected at any time. Obviously, these organisms were practically eliminated by the E-beam treatment (data not shown).

### 3.3. Food Safety Aspects

Accidental pathogen contamination during the marinated loin slice preparation is a phenomenon that affects the slice surfaces and, on the other hand, the marinated pork loin is a product intended to be eaten once it is cooked. Consequently, the risk of pathogen organisms will be eliminated during the cooking process. Nevertheless, in an attempt to reach the highest hygienic status, many countries regulate the presence of *Salmonella*. For example, the European Community (EC no. 1441/2007) stipulates the safety criterion of absence in 10 g of products placed on the market during their entire shelf-life for *Salmonella* in “minced meat and meat preparations made from other species than poultry intended to be eaten.” Furthermore, if a temperature abuse occurs, it is also possible that *Salmonella* spp., and other pathogen bacteria (e.g., *S. aureus*), unable to grow at 4°C but able to grow at >6–8°C could multiply if present. No regulation has been set by the EC for *L. monocytogenes* in relation to this kind of product. However, as the shelf-life is extended by E-beam treatment, there is, if present, an opportunity for *L. monocytogenes* to grow due to its psychrotrophic condition thereby increasing the risk of dissemination of this organism through cross-contamination. Therefore, it seems convenient to be aware of its potential increase in numbers during the storage period. When trying to optimize any process (in this case E-beam treatment) to sanitize a meat product, taking into account the growth of *L. monocytogenes* during its shelf-life is necessary in order to eliminate it to ensure a level that guarantees the product safety. The resulting treatment will be enough to reduce the number of *Salmonella*, if present, since the latter bacterium does not grow (at 4°C) or grow more slowly (at 8°C) than *L. monocytogenes* [26]. In fact, the previous literature reports a *g*-value of 22 h at 10°C for *S*. *Enteritidis* [75] and 5–7 h at 9.3°C for *L. monocytogenes* [76]. For these reasons, in this work the optimization with E-beam treatment has been performed with *L. monocytogenes*, using *L. innocua* as a surrogate*.*

The response of *L. innocua* to the E-beam treatment was fitted to first-order inactivation kinetics, following the equation: log CFU/cm^2^ = 7.0277 − 2.1918 × Dose (*R* ^2^ = 0.9944), from which a decimal reduction value (*D*-value) of 0.46 kGy was calculated. This value validate the death kinetic of this bacterium in meat products since values of 0.49 kGy and 0.44 kGy were previously determined in cooked ham [34] and fresh pork loin [30]. Among nonsporeforming pathogens, *L. monocytogenes* is one of the most radioresistant bacterium [62, 63, 77, 78].

Several authors have reported increased numbers of *L. monocytogenes* in various products stored at 4-5°C. The data have been collected in a FDA report [79] from which an average increase of 0.2 log units/day may be estimated in fresh meat and 0.35 log units/day when storage is at 8°C. So, assuming a contamination in the raw marinated loin of 10 cells/cm^2^ (log = 1), as suggested by the ICMSF [80], the load of the nonirradiated loin slices will be 250 CFU/cm^2^ at 4°C at the end of shelf-life (7 days) and 562 CFU/cm^2^ at 8°C (shelf-life of 5 days). Nevertheless, E-beam treatment provokes a 2.17 D and 4.35 D reduction with the application of 1 and 2 kGy, respectively. Therefore, the E-beam treatment will reduce the level of listeria to 6.76 × 10^−2^ CFU/cm^2^ and 4.46 × 10^−4^ CFU/cm^2^, respectively. As this bacterium is able to grow under refrigeration, its numbers will increase during storage in such a way that, assuming the same growth rates, the levels will be around 107 CFU/cm^2^ at the end of the shelf-life at 4°C (16 days) with a dose of 1 kGy and 4.57 CFU/cm^2^ if 2 kGy was applied. In a temperature abuse situation (8°C), with similar reasoning, the *L. monocytogenes* load at the end of the shelf-life may be estimated in 214 CFU/cm^2^ and 182 CFU/cm^2^. Thus, the E-beam treatment has led to an important improvement of the hygienic status.

Although more than 2,000 serovars of *Salmonella enterica* are known, most infections in humans are caused by only a few serovars, the most common of which corresponds to *S*. *enteritidis* and* S*. *typhimurium* [81]. Publications [37, 61, 82] have repeatedly confirmed that the resistance of *S. typhimurium* to irradiation is significantly higher than that of *S*. *enteritidis*, and the *D*-value for the former organism is in the level of 0.45–0.50 kGy. Assuming a contamination similar to that of *L. monocytogenes* (i.e., 10 CFU/cm^2^) and a *D*-value of 0.47 kGy for *S*. *typhimurium*, the application of 1 kGy or 2 kGy would reduce the number of *Salmonella* 2.13 D and 4.25 D, respectively. These figures mean that *Salmonella* loads posttreatment would be <1 CFU/10 cm^2^ and <0.01 CFU/10 cm^2^. These numbers do not increase at 4°C and thus, the EC microbial regulation for *Salmonella* spp. is complied. Furthermore, it has been reported in cooked ham [36] that the growth of *L. monocytogenes* after the E-beam treatment is significantly decelerated, increasing the lag and the exponential phases, which suggests that surviving organisms are not able to grow to a dangerous level. In general, it could be concluded that consumer health is safeguarded and cross-contamination minimized.

### 3.4. Color, Textural, and Sensory Measurements

#### 3.4.1. Instrumental Color

**Figure 3:**
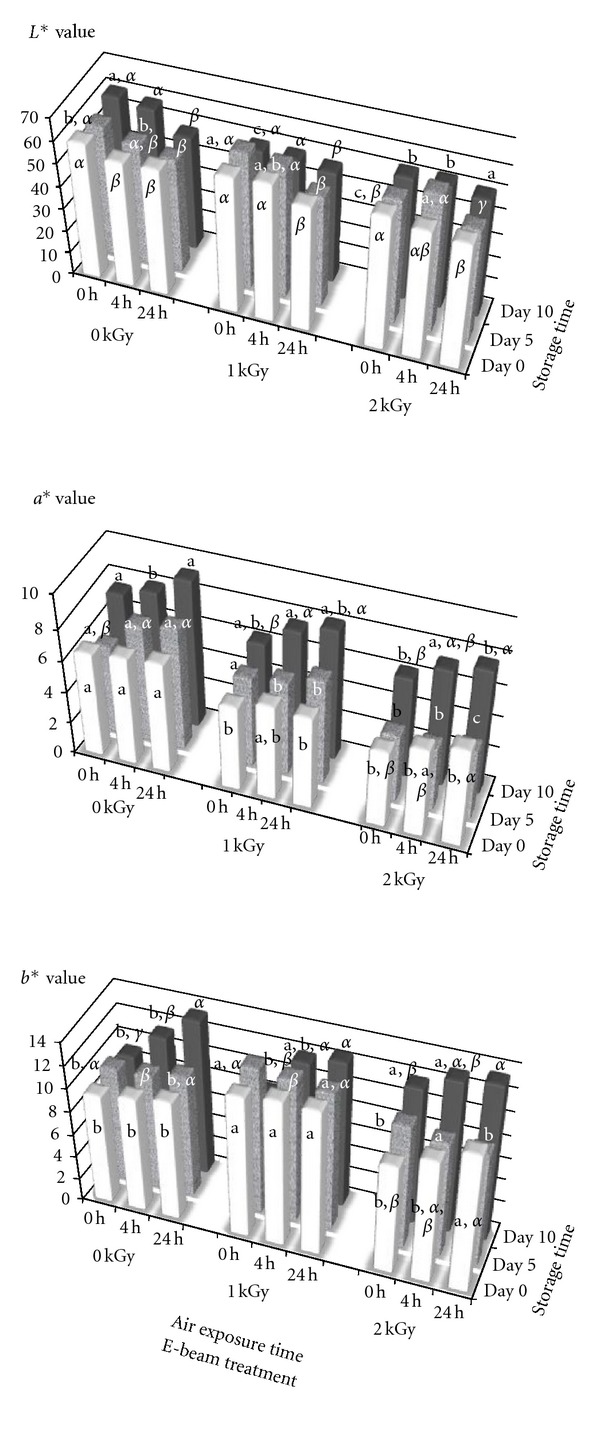
Effect of several factors (E-beam treatment, air exposure, and storage times) on the color parameters (L*, a*, and b*). a, b, c in column values for storage and air exposure times with different letters are significant differences (*P* < 0.05). *α*, *β*, *γ* in rows values for the treatment dose and storage time with different letter are significant different (*P* < 0.05).

The marinated loin is a product that is intended to be eaten once it has been cooked, therefore, the flavor, color and, in general, the appearance will change during the cooking process. Nevertheless, the color is, perhaps, the most important feature since the packaged product is displayed in the refrigerated cabinet shelves waiting to be chosen by the consumers.  Figure 3 shows the results of the instrumental measurements of the marinated loin slice color in samples stored at 4°C. Data related to 10th day of storage of nonirradiated samples are not considered because their shelf-life ended the 7th day (Table 2) and by the 10th day the sample surface would be coated with the slime produced by Gram-negative microbiota [83]. Beside this, overall, no great differences were found, the a* (redness) being the most affected value (Figure 3). Just after the treatment, a slight decrease (about 10–12%) of the value of this parameter was observed. However, the a* value rose (*P* > 0.05) with increased storage irrespective of the dose applied. In addition, in each sample (treated with 0, 1, or 2 kGy and stored at 4°C postprocessed) this parameter increased with more time of air-exposure once the package was opened. All these differences were minimized as the storage was extended in such a way that after 10 days, regardless of the dose, the E-beam treated samples at the beginning (0 day to the air exposure) were slightly more red but after exposure to air for 4 or 24 h the differences were practically eliminated. In the fresh loin [30], the differences in the a*, b*, and L* parameters between control and E-beam treated samples were clearer than in marinated loin. It could be attributed to the marinating substances (mainly the paprika) overlooked the oxidizing effect of the radiation, which may be due to the availability of oxygen during the E-beam treatment. Besides this, free radicals, ozone [84], and oxygen peroxide [85] are produced by radiolysis of water. These compounds are strong oxidizing agents which, in turn, could work together with the oxygen to oxidize several meat compounds, in this case the myoglobin (red), yielding traces of metmyioglobin (brown) responsible for the deeper red color of the meat. Minor changes were detected in the parameters L* and b* (Figure 3). All values were similar except those that corresponded to the control samples of 10 days, already discussed previously (spoiled on the 7th day) and in those treated with 2 kGy, in which the increase of the yellowness could be noted in samples stored for 10 days. 

#### 3.4.2. Textural and Breaking Strength

The effects of the E-beam application (1 and 2 kGy) on selected textural attributes (hardness, adhesiveness, springiness, cohesiveness, gumminess, chewiness, and the breaking strength) were explored. Since this product was conceived to be eaten after being cooked, the former attributes are of less concern. Briefly, no differences were found in adhesiveness, springiness, and breaking strength between control samples and those treated by E-beam. A significant difference (*P* < 0.05) was observed in the dimensionless cohesiveness (average of 6 measurements ± standard) between samples treated with 0 and 1 kGy (0.51 ± 0.11 and 0.53 ± 0.16, resp.) and those treated with 2 kGy (0.65 ± 0.21). The major significant difference (*P* < 0.05) was found in the hardness (average of 6 measurements ± standard deviation) following the treatment and just after opening the bags, which was softer in control samples (21.85 ± 6.54 N) than those treated at 1 (35.83 ± 5.5 N) and 2 kGy (35.08 ± 6.23 N) although these differences disappeared after 5 days of storage at 4 or 8°C, when similar values to the control samples were achieved (average of 29.90 ± 8.95 N). Obviously, significant differences (*P* < 0.05) were also detected in the secondary attributes (gumminess and chewiness) related to the hardness, but they disappeared after storage for a few days. In [54], an increase in hardness of cooked chicken breast treated at 2.9 kGy, due to shrinkage of the myofibrils was also reported. However, reports show that E-beam treatments at doses lower than 3 kGy did not affect the textural features of several meat products, including turkey beast rolls [86], cooked ham [34], dry ham [87], and fermented sausages [35]. These opposed differences may occur because pork loin is a raw meat, whereas the former items are transformed meat products with a higher dry matter content and, therefore, they present a more robust texture that may be less sensitive to physical technologies, such as irradiation and light pulse [37, 88]. During storage, the only clear difference observed affected adhesiveness, which increased with a longer storage time (*P* < 0.05), which is probably related with the formation of slime by the Gram-negative bacteria [69, 74]. Slight differences were occasionally found for some of the remaining attributes, but they did not follow a consistent pattern. Perhaps, the clearest one was the decrease in hardness in some samples regardless of the treatment applied, which has been attributed to the activity of endogenous proteinases.

#### 3.4.3. Sensorial Aspects

The effects of E-beam treatment on the sensory attributes of marinated pork loin stored at 4°C was evaluated by triangular, rank order, and descriptive tests. As determined by both the triangular and the rank order tests, significant differences were obtained (*P* < 0.05) for appearance and odor in untreated (0 kGy) and E-beam treated (1 and 2 kGy) samples, immediately after treatment (0 days) and after storage at 4°C (7 days). In the descriptive analysis, the appearance of samples immediately after the E-beam and after 7 days of storage was considered to be similar in appearance to the control samples, which was in agreement with the instrumental color measurements (Figure 3). Nevertheless, at 2 kGy they were judged to be pale pink, slightly reddish-brown. However, these samples were also considered acceptable for trading. Moreover, in the descriptive analysis carried out at the end of the shelf-life of the E-beam treated samples (11 and 20 days at 1 and 2 kGy, resp.) similar color features to those mentioned above were described. Additionally, it is noteworthy, that there were no significant differences (*P* > 0.05) for appearance between untreated and treated samples when they were cooked.

In relation to the odor, in the descriptive analysis, both immediately after treatment (0 days) and after 7 days of storage at 4°C, in the raw samples treated at 1 kGy, the typical fresh marinating odor was slightly lost and a negligible odor like “scalded feather” was detected. This odor was clearer in the samples treated at 2 kGy and, additionally, slight off-odor defined as “scalded feather,” “poultry,” metallic and sulfur taints were identified. These off-odors were detected when samples were air exposed after opening the packaged bags. More than 7% of the volatiles found in irradiated foods are hydrocarbons commonly found in thermally processed and unprocessed foods [89]. Most chemical changes in irradiated meat are associated with free radical reactions [90]. The off-odors detected in the E-beam treated samples would be responsible for the lower scores assigned to the treated samples versus those that were untreated (data not shown) in the rank order text. Despite this effect, the radiated samples, even at 2 kGy, were qualified as acceptable for trading. Moreover, after 7 days of storage at 4°C, the untreated raw samples showed a slight off-odor associated with the growth of spoilage organisms and the aging of meat (pungent, sour, unpleasant). Irradiation can slightly increase levels of dimethyl disulfide, dimethyl trisulfide, S-methyl ester, and ethanoic acid. These sulfur compounds are highly volatile and can be eliminated by storing the irradiated meat under aerobic conditions [91]. After cooking, a slight off-odor was detected only in the samples treated at 2 kGy. These results are in agreement with the findings of other authors, who reported that cooking can reduce or eliminate irradiation-induced odor [92, 93].

In cooked samples, the flavor analysis by both the triangular and the rank order tests, significant differences (*P* < 0.05) were only found when untreated and treated samples at 2 kGy were compared just after E-beam application (data not shown). In the descriptive analysis, samples treated at 2 kGy were judged to be less juicy and had a very slight taint of “burnt,” “hot culture medium,” acids and metallic notes and negligible, astringent feel aftertaste. It has been reported [94, 95] that postirradiation storage can allow flavor to return to the near normal features of the untreated products as the volatiles are lost. Much of the work on irradiated meat odor and flavor has targeted selected constituents, particularly lipids [96, 97]. The reactions of sulfur-containing amino acids with water radiolytic products appear to be the source of hydrogen sulfide and other volatile sulfur-containing compounds which contribute to off-flavor [98]. The literature reports that irradiation [94] increases the concentration of 3-methylbutanal and 2-methylbutanal, mainly in vacuum packaged samples. However, dimethyl disulphide levels did not differ between irradiated and untreated samples in aerobic packaging [91]. In irradiated cooked meat, a slightly higher volatile content has been found than in irradiated meat that was subsequently cooked [91].

## 4. Conclusions

The shelf-life of marinated pork loin slices at 4°C may be extended from 7 to 16 and 20 days with the application of 1 and 2 kGy, respectively. Likewise, there is a mild temperature abuse (increase to 8°C), the shelf-life will be extended from 5 to 10 and 16 days, respectively, when applying the same dose without compromising the sensory quality. From a hygienic point of view, E-beam treated marinated loin that is stored under refrigeration (4°C) practically guarantees a pathogen-free product during its shelf-life. Minor changes are produced in the main sensory characteristics, including the flavor of the coked product.

## Figures and Tables

**Table 1:** Effect of E-beam treatment on the water holding capacity (WHC) of marinated pork loin following treatment and 8 days of storage at 4 and 8°C.

Doses	Day 0	Day 8 (4°C)	Day 8 (8°C)
0 kGy	41.37 ± 2.7^b^	54.32 ± 1.8^a^	51.95 ± 0.99^a^
1 kGy	51.37 ± 8.15^a^	36.64 ± 6.9^b^	34.34 ± 6.48^b^
2 kGy	48.32 ± 1.78^a^	43.51 ± 3.2^b^	33.06 ± 7.17^b^

^a,b^Values at the same column with different letters indicate significant differences (*P* < 0.05).

**Table 2:** Shelf-lives at 4 and 8°C of fresh and marinated pork loin subjected to E-beam treatment.

Dose (kGy)	Fresh*	Marinated	Fresh*	Marinated
	4°C	4°C	8°C	8°C
0	5	7	3	5
1	11	16	8	10
2	20	>20	16	16

*Data from [30]. Shelf-life was established when the total viable counts exceeded 5 × 10^7^ CFU/cm^2^.
